# Photodynamic therapy for bullous retinal detachment: a single-center experience of case series with a 6-month follow-up study

**DOI:** 10.1007/s00417-018-4015-8

**Published:** 2018-06-04

**Authors:** Tingting Gao, Jinfeng Qu, Jing Xiao, Jie Hu, Mingwei Zhao

**Affiliations:** 1 Department of Ophthalmology, Eye Diseases and Optometry Institute, Beijing Key Laboratory of Diagnosis and Therapy of Retinal and Choroid Diseases, College of Optometry, Peking University People’s Hospital, Peking University Health Science Center, Xizhimen South Street 11, Xi Cheng District, Beijing, 100044 China; 2 Department of Ophthalmology, 3rd People’s Hospital of Linyi, Linyi, China

**Keywords:** Bullous retinal detachment, Efficacy, Photodynamic therapy, Multifocal, Large laser spot

## Abstract

**Purpose:**

To evaluate the efficacy of half-dose photodynamic therapy (PDT) for the treatment of bullous retinal detachment.

**Methods:**

Interventional prospective case series in six eyes from six consecutive patients with bullous retinal detachment. The effected eyes were treated with indocyanine green angiography (ICGA)-guided half-dose PDT with multifocal and large laser spots. Clinical evaluations included best-corrected visual acuity (BCVA), ophthalmoscopy, ophthalmic B scan, fundus fluorescein angiography (FFA), optical coherence tomography (OCT), and ICGA at each scheduled visit at baseline; at 1, 3, and 6 months after PDT; and during follow-up after 6 months.

**Results:**

All six eyes received half-dose verteporfin PDT with a mean number of therapeutic spots 2.83 ± 1.47 and a mean spot size of 4647 ± 996 μm in diameter. Three months after PDT, retinal reattachment was observed on B scans and resolution of sub-retinal fluid (SRF) was observed in OCT images for five eyes. There was no significant difference in the mean logMAR BCVA between the baseline and the value at 1 month after PDT (*P* = 0.477). At 3 months after PDT, the mean logMAR BCVA improved significantly from a baseline value of 1.02 to 0.54 (*P* = 0.044). At 6 months after PDT, the mean logMAR BCVA further improved to 0.46 (*P* = 0.025) and remained stable. One affected eye received a second half-dose PDT for SRF not reduced until the second month after PDT. Retinal reattachment and SRF resolution were observed at 1 and 3 months after the second therapy, respectively. BCVA improved from a baseline value of 20/63 to 20/20 at 1 month after the second PDT and remained stable until the sixth month after the second PDT. During follow-up after more than 6 months, recurrence occurred in no cases.

**Conclusions:**

This study demonstrated half-dose PDT with multifocal and large laser spots was an effective treatment for bullous retinal detachment contributing to the retinal reattachment, a resolution of SRF, and an improvement of BCVA. Thus, PDT for the treatment of bullous retinal detachment is considered to be a worthwhile endeavor.

## Introduction

Chronic central serous chorioretinopathy (CSC) is a major threat to central vision in the working population and is mainly characterized by the serous detachment of the neurosensory layer of the retina from the retinal pigment epithelium in the macular area [1, 2]. The detachment spontaneously resolves within 2 to 3 months in two-thirds of affected individuals, which is defined as acute CSC [3]. However, more than one-third of patients experience chronic forms of CSC with persistent sub-retinal fluid (SRF) accumulation or recurrences leading to continuous visual impairment [4]. The vascular leakage of chronic CSC results from the dysfunction of the retinal pigment epithelium (RPE) outer blood-retinal barrier, most likely caused by choroidal vasodilatation and hyperpermeability [5, 6]. Bullous retinal detachment, which can eventually lead to vision loss as a result of irreversible retinal damage, is an extremely rare manifestation of CSC. However, the precise pathogenesis of why only a small subset of eyes with CSC are complicated by bullous retinal detachment has remained unclear until now. In recent decades, the relationship between CSC and corticoid has been one of the most intriguing aspects of the disease. A previous study reported that glucocorticoids can reduce macular edema and aggravate SRF accumulation in CSC patients [6]. Additionally, in previous reports, the occurrence of bullous retinal detachment was also associated with a history of glucocorticoids [7, 8].

Based on the currently available literature, the traditional treatment of CSC consists of photodynamic therapy (PDT) and laser photocoagulation. However, there are limited clinical data on the therapy of patients with bullous retinal detachment thus far. In the present study, we present a series of cases of bullous retinal detachment that responded favorably to half-dose PDT without any additional complications during the follow-up period of more than 6 months.

## Methods

### Patients

Six eyes of six patients with bullous retinal detachment secondary to CSC were included in this prospective study. The diagnosis of bullous retinal detachment was established by ophthalmoscopy, ophthalmic B scan, fundus fluorescein angiography (FFA), optical coherence tomography (OCT), and indocyanine green angiography (ICGA). Bullous retinal detachment was designed as a neurosensory detachment (more than 10 disc diameters) with shifting SRF attributed to a leak or leaks at the level of the RPE with a bullous appearance that extended to the inferior vascular arcades [9]. All of the following had to be present to diagnose bullous retinal detachment: exudative retinopathy observed by fundoscopy, retinal detachment observed by B scan, SRF observed by OCT, vascular leakage observed by FFA, and corresponding hyperfluorescence observed by ICGA. The patients were consecutively enrolled from July 2015 to May 2017 in Peking University People’s Hospital. The patients recruited received PDT without other kind of treatments. The study protocol was approved by the Ethical Committee and Institutional Review Board of Peking University People’s Hospital (Beijing, China) and was in accordance with the Declaration of Helsinki. Written informed consent was obtained from the recruited bullous retinal detachment patients prior to PDT.

### Photodynamic therapy treatment and follow-up

Half-dose intravenous (3 mg/m^2^) verteporfin was infused over a period of 10 min. A contact glass was positioned on the affected eye, followed by delivery of 689 nm laser energy to the target zone 15 min after infusion. Abnormal vascular areas selected based on hyperfluorescent zones observed by ICGA of the mid-phase, corresponding to hyperfluorescent leakage on FFA of the mid-phase were treated. For PDT, the spot sizes were chosen according to abnormal vascular areas and a total light energy of 50 J/cm^2^ for 83 s was used. Separate areas of choroidal hyperpermeability were treated separately. If SRF was not reduced until the second month after PDT, then the eye received another half-dose PDT. The patients were instructed to avoid light for 48 h after PDT. BCVA, ophthalmoscopy, ophthalmic B scan, FFA, OCT, and ICGA results were examined upon follow-up at 1, 3, and 6 months after treatment.

### Statistical analyses

For statistical analyses, a paired-samples *t* test was used in SPSS Statistics (version 20; IBM) to compare logMAR BCVA during each visit at baseline and 1, 3, and 6 months after PDT. The level of statistical significance was set at *p* < 0.05.

## Results

This study included six eyes from six consecutive patients. Table 1 summarizes the clinical characteristics of six patients with bullous retinal detachment. The patients were five males and one female with an average age of 40.5 ± 6.7 years (mean ± standard deviation). The disease affected a single eye in all six patients (100%). Regarding medical history, two patients (33.3%) had bullous retinal detachment associated with systematic corticosteroid therapy for initial nephritic syndrome and intravitreal injection of triamcinolone acetonide (TA) for misdiagnosis of uveitis. Four patients (66.7%) presented with characteristics of classic CSC initially, followed by development of bullous retinal detachment 3 months to 10 years later. Except for one patient who lost vision in the fellow eye because of glaucoma at a young age, the remaining five patients had classic CSC in the fellow eyes.

**Table 1:** Clinical information of six patients with bullous retinal detachment

Case no.	Sex	Age at onset, year	Eyes affected	History of glucosteroid	Treatments	Total number of PDT spots	Mean diameter of spots, μm	Retinal reattachment post-PDT on B scan, months	Resolution of SRF on OCT, months	BCVA of baseline	BCVA of the 6th month after PDT	Recurrence?
1	F	35	RE	Oral prednisone for 6 years	50% PDT	1	5000	3	3	20/63	20/50	No
2	M	37	LE	No	50% PDT	2	4500	3	3	20/200	20/40	No
3	M	38	RE	No	Twice 50% PDT	4	4500	1 month after the 2nd PDT	3 months after the 2nd PDT	20/63	20/20	No
4	M	48	LE	Intravitreal injection of TA a month before	50% PDT	3	4333	3	3	20/200	20/100	No
5	M	50	RE	No	50% PDT	5	4800	3	3	20/200	20/40	No
6	M	35	LE	No	50% PDT	2	5000	3	3	20/800	20/80	No

*PDT* photodynamic therapy, *SRF* sub-retinal fluid, *RE* right eye, *LE* left eye, *TA* triamcinolone acetonide

Bullous retinal detachment was invariable in the posterior pole and peripheral retina, characterized by accumulation of SRF shifting on different positions. Retinal detachment and macular SRF were observed in six eyes (100%). Peripheral hyperfluorescent foci were observed by FFA in five eyes (83.3%). Sub-retinal fibrin occurred in three eyes (50%) as observed by OCT. Two eyes (33.3%) were identified with retinal folds.

**Fig. 1:**
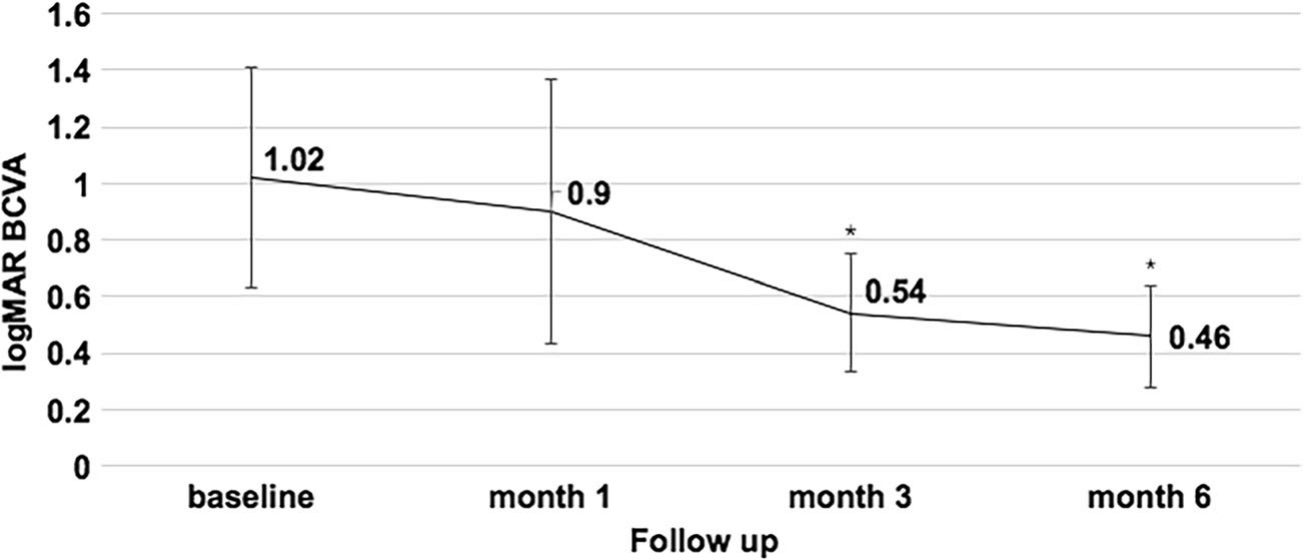
Time course of changes in best-corrected visual acuity (logMAR) of five eyes received single 50% dose PDT. Error bars = standard errors. ^*^P < 0.05

All six eyes received ICGA-guided half-dose verteporfin PDT. The mean number of PDT spots was 2.83 ± 1.47 (range from 1 to 5) with a mean spot size of 4647 ± 996 μm in diameter (range from 3000 to 6000 μm). Three months after PDT, retinal reattachment on B scan and resolution of sub-retinal fluid (SRF) on an OCT image were observed in five eyes. There was no significant difference in the mean logMAR BCVA between the baseline and 1 month after PDT (*P* = 0.477). At 3 months after PDT, the mean logMAR BCVA improved significantly from a baseline value of 1.02 to 0.54 (*P* = 0.044). At 6 months after PDT, the mean logMAR BCVA further improved to 0.46 (*P* = 0.025) and remained stable. The changes in BCVA of the five eyes at different time points are shown in Fig. 1. One affected eye (16.7%) received a second half-dose PDT, as SRF was not reduced until the second month after the first therapy. Next, we adjusted the therapeutic areas of PDT based on ICGA imaging. Retinal reattachment was observed 1 month after the second therapy, and SRF resolution was observed 3 months after the second therapy. BCVA improved from a baseline value of 20/63 to 20/20 1 month after the second PDT and remained stable until the 6th month after the second PDT.

No major sequelae for PDT, such as persistent choroidal ischemia, macular hemorrhage, or severe chorioretinal degeneration, were observed in any eyes during the follow-up periods. During the follow-up more than 6 months, no recurrence occurred.

### Case reports

#### Case 1

**Fig. 2:**
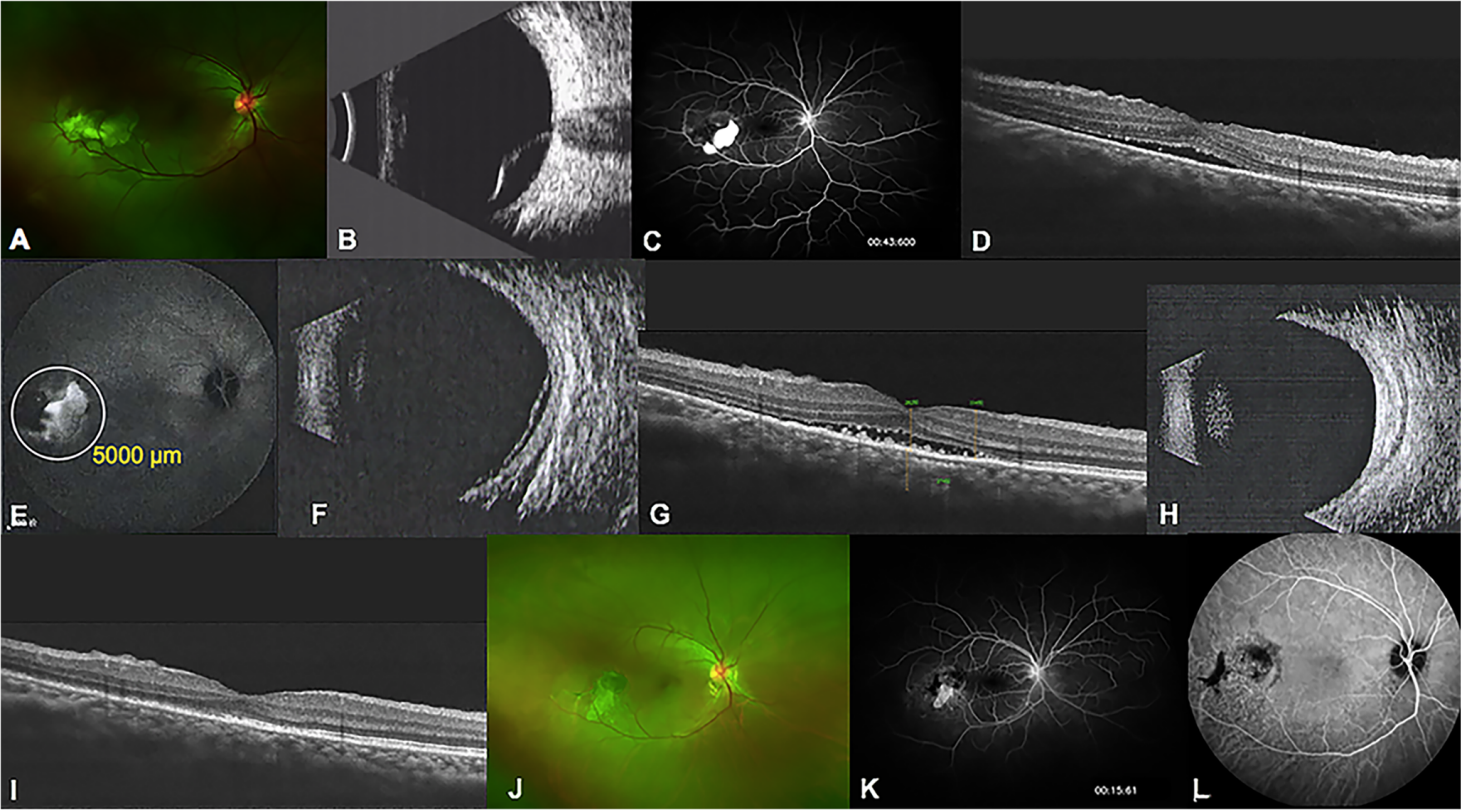
Clinical examinations of the right eye in case 1. **a** Fundus photograph taken on the initial presentation illustrating bullous retinal detachment in the posterior pole. **b** Ophthalmic B scan revealing bullous retinal detachment at baseline. **c** FFA image demonstrating hyperfluorescent leakage in areas corresponding to exudative retinal detachment at baseline. **d** OCT image showing the neuro-retinal detachment in macula at baseline. **e** ICGA image illustrating hyperfluorescent leakage of choroid at baseline. White circle indicates the spot size of PDT. **f** Ophthalmic B scan revealing bullous retinal detachment partly resolved at 1 month after PDT. **g** OCT image showing deposits at the area of detachment especially on RPE during the course of SRF reducing at 1 month after PDT. **h** Ophthalmic B scan revealing reattachment of retina 3 months after PDT. **i** OCT image showing the disappearance of SRF 3 months after PDT. **j** Fundus photograph obtained 6 months after PDT, illustrating the mottled appearance in the posterior pole. **k** FFA image showing a mottled shape in fluorescence transmission and no fluorescein leakage 6 months after PDT. **l** ICGA image showing hypofluorescence in the lesion area 6 months after PDT

A 35-year-old woman presented with blurred vision and photopsia in both eyes for 3 months. Her BCVA was 20/63 OU (in each eye). Ophthalmoscopy and ophthalmic B scan of the right eye revealed bullous retinal detachment in the posterior pole (Fig. 2a, b). The patient was treated with oral prednisone at 30 mg per day for over 6 years for nephritic syndrome. FFA of the right eye revealed hyperfluorescent leakage in areas corresponding to exudative retinal detachment (Fig. 2c). OCT of the right eye disclosed neuro-retinal detachment (Fig. 2d). The left eye had focal retinal pigment epithelial detachment in the inferior retina. The right eye received a 50% dose of verteporfin PDT with a spot size of 5000 μm to cover the abnormal vascular area in indocyanine green angiography (Fig. 2e). One month later, the SRF was partly reduced (Fig. 2f, g), while 3 months later, there was retinal reattachment, and the complete absence of SRF was confirmed by ophthalmic B scan and OCT (Fig. 2h, i). At the 6-month examination after photodynamic therapy, the BCVA improved to 20/50 in the right eye, and a mottled appearance was observed in the fundus (Fig. 2j). FFA showed a mottled shape upon fluorescence transmission, and no fluorescein leakage was observed (Fig. 2k). ICGA showed hypofluorescence in the lesion area (Fig. 2l).

#### Case 2

**Fig. 3:**
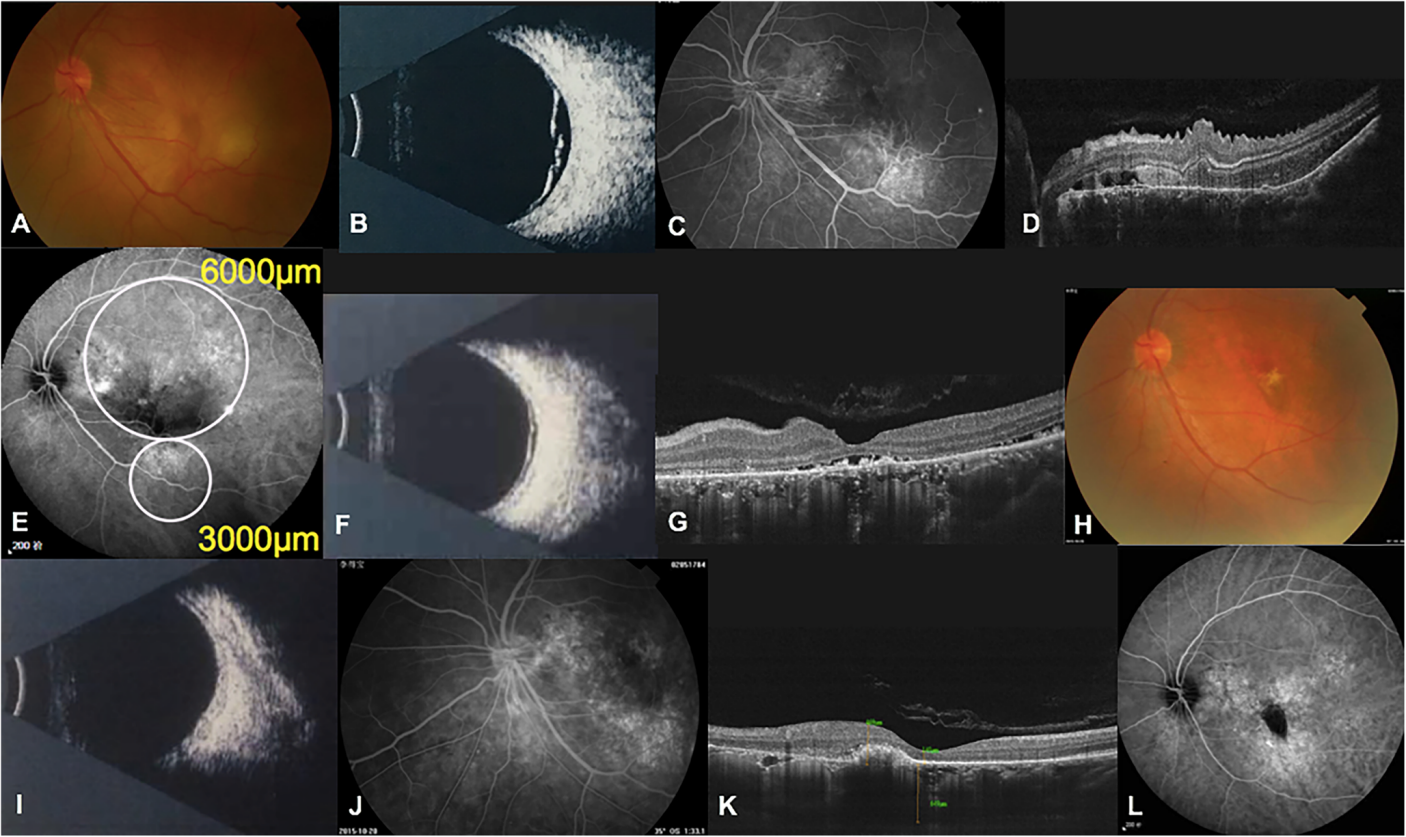
Clinical examinations of the left eye in patient 2. **a** Fundus photograph disclosing exudative lesions in the macula and inferotemporal retina at baseline. **b** Ophthalmic B scan revealing bullous retinal detachment at baseline. **c** FFA image revealing hyperfluorescent leakages in areas of exudative retinal detachment at baseline. **d** OCT image disclosing SRF, sub-retinal fibrin and retinal folds at baseline. **e** ICGA image illustrating several hyperfluorescent leakages of the choroid at baseline. White circles indicate the spot sizes of PDT. **f** Ophthalmic B scan revealing obvious resolution of the bullous retinal detachment 1 month after PDT. **g** OCT image disclosing obvious reduction in SRF and disappearance of sub-retinal fibrin 1 month after PDT. **h** Fundus photograph taken 3 months after PDT illustrating the disappearance of the exudative lesions. **i** Ophthalmic B scan revealing the retinal reattachment 3 months after PDT. **j** FFA revealing the mottled appearance of transmitted fluorescence and no hyperfluorescent leakage 3 months after PDT. **k** OCT showing complete resolution of SRF and the disappearance of retinal folds 3 months after PDT. **l** ICGA image showing the disappearance of hyperfluorescent leakage and mottled appearance remaining 3 months after PDT

A 35-year-old man first noted visual loss in both eyes and was diagnosed with classic central serous chorioretinopathy; he received argon laser photocoagulation in the right eye at another hospital. Two years later at the age of 37, the patient experienced severe blurring in the left eye, with a BCVA of 20/80 in the right eye and 20/200 in the left eye. Ophthalmoscopy of the left eye disclosed exudative lesions in the macula and inferotemporal retina (Fig. 3a), which were confirmed as bullous retinal detachment by ophthalmic B scan (Fig. 3b). FFA revealed hyperfluorescent leakage in areas of exudative retinal detachment (Fig. 3c). OCT disclosed SRF, sub-retinal fibrin adjacent, and retinal folds in the lesion area (Fig. 3d). His left eye received a 50% dose of verteporfin PDT with two spots of 6000 and 3000 μm under the guidance of ICGA (Fig. 3e). One month after PDT, the SRF obviously decreased and sub-retinal fibrin disappeared based on ophthalmic B scan and OCT (Fig. 3f, g). Three months later, ophthalmoscopy and ophthalmic B scan showed that the exudative retinal detachment was resolved completely at the macula (Fig. 3h, i). FFA revealed a mottled appearance of transmitted fluorescence and no hyperfluorescent leakage (Fig. 3j), and OCT showed the complete resolution of SRF and the disappearance of retinal folds (Fig. 3k). ICGA also disclosed the disappearance of hyperfluorescent leakage and the mottled appearance that remained (Fig. 3l). At the 6-month examination, his BCVA improved to 20/40 in the left eye and remained stable.

#### Case 3

**Fig. 4:**
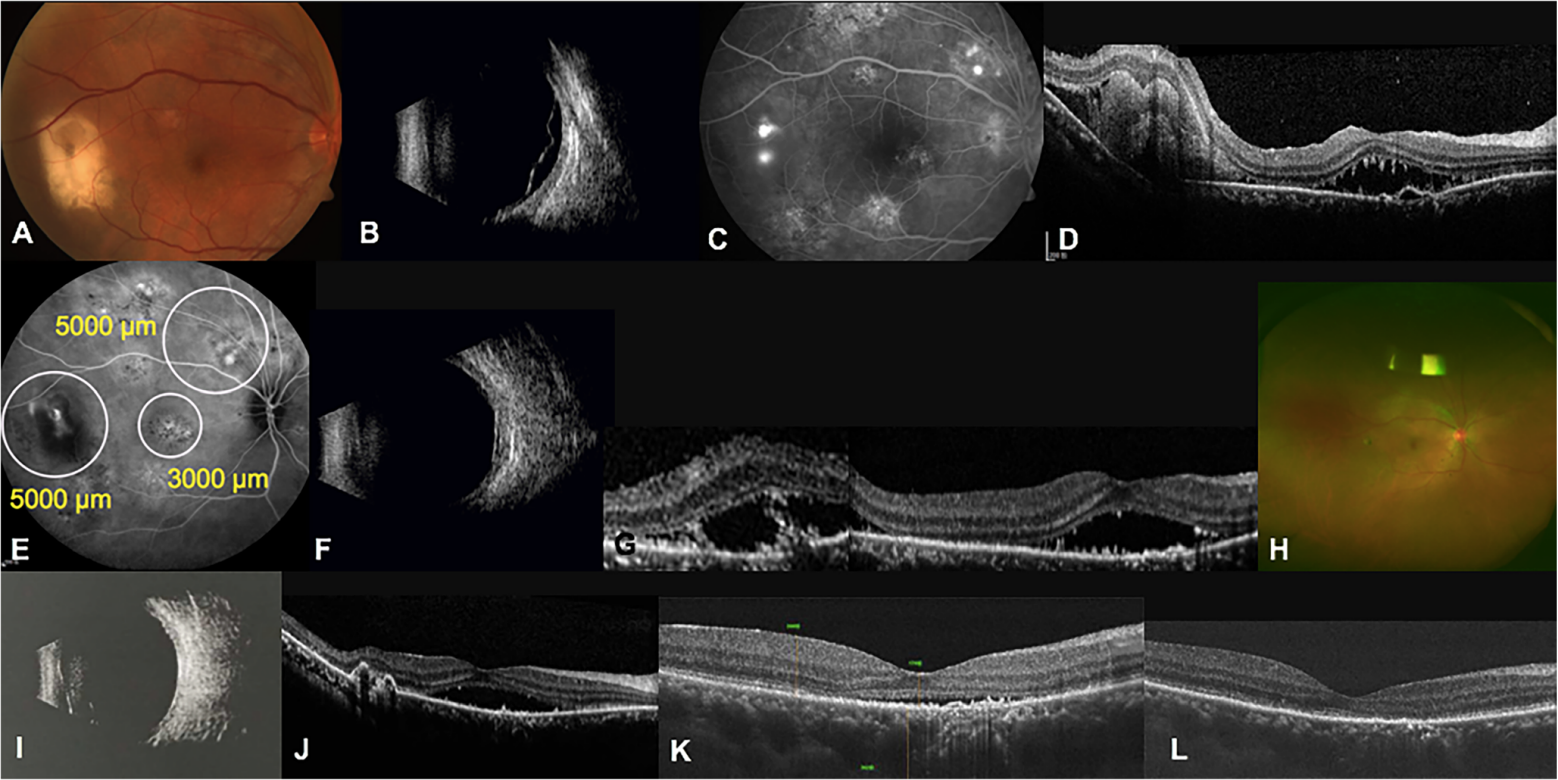
Clinical examinations of the right eye in patient 3. **a** Fundus photograph taken at the initial presentation revealing retinal detachment accompanied with yellowish, fibrinoid exudative lesions in the temporal of macula. **b** Ophthalmic B scan revealing bullous retinal detachment at baseline. **c** FFA image revealing several hyperfluorescent leakages at baseline. **d** OCT image disclosing severe SRF and sub-retinal fibrin at baseline. **e** ICGA image illustrating several hyperfluorescent leakages of the choroid at baseline. White circles indicate the spot sizes of PDT. **f** Ophthalmic B scan disclosing an obvious reduction in bullous retinal detachment 1 month after PDT. **g** OCT image revealing that the SRF slowly resolved with reduced sub-retinal fibrin 2 months after PDT. **h** One month after the second PDT (3 months after the first PDT), a fundus photograph revealed the disappearance of yellowish, fibrinoid exudative lesions. **i** Ophthalmic B scan revealing the reattachment of retina 1 month after the second PDT. **j** OCT image showing markedly decreased SRF in the temporal area 1 month after the second PDT. **k** OCT image revealing nearly resolved SRF 2 months after the second PDT. **l** OCT image showing the complete resolution of SRF 3 months after the second PDT

A 38-year-old man with a history of blurred vision in the right eye for 1 year was diagnosed with CSC in the left eye, which was treated with argon laser photocoagulation 10 years ago. His BCVA was 20/63 in the right eye and 20/200 in the left eye. Ophthalmoscopy of the right eye revealed retinal detachment accompanied with yellowish, fibrinoid exudative lesions in the temporal macula (Fig. 4a). Ophthalmic B scan confirmed bullous retinal detachment in the right eye (Fig. 4b). FFA disclosed multiple intense sub-retinal leakage in areas corresponding to exudative lesion and in the superior retina (Fig. 4c). OCT disclosed large amounts of SRF in the macular and temporal retinal areas (Fig. 4d). His right eye received a 50% dose of verteporfin PDT with three spots under the guidance of ICGA (Fig. 4e). Ophthalmic B scan disclosed obviously reduced bullous retinal detachment 1 month after PDT (Fig. 4f), and his BCVA improved to 20/25. However, the SRF slowly resolved. Although the OCT image revealed a reduction in sub-retinal fibrin, the SRF remained and resolved slowly until the second month after PDT (Fig. 4g). Considering the slow resolution of SRF, the patient received a second 50% dose of verteporfin PDT in the right eye. We adjusted the therapeutic area with a spot size of 5000 μm under the guidance of ICGA. One month after the second PDT (3 months after the first PDT), ophthalmoscopy of the right eye revealed the disappearance of the yellowish, fibrinoid exudative lesions (Fig. 4h). Additionally, the reattached retina was observed on B scan, and SRF in the temporal area markedly decreased in the OCT image (Fig. 4i, j). OCT revealed nearly resolved SRF 2 months after the second PDT (Fig. 4k), and the SRF was completely resolved 3 months after the second PDT (Fig. 4l). The patient’s BCVA of the right eye improved to 20/20 in the third month after the second PDT and remained stable until the sixth month after the second PDT. No recurrence occurred during the follow-up of more than 6 months after the second PDT.

#### Case 4

**Fig. 5:**
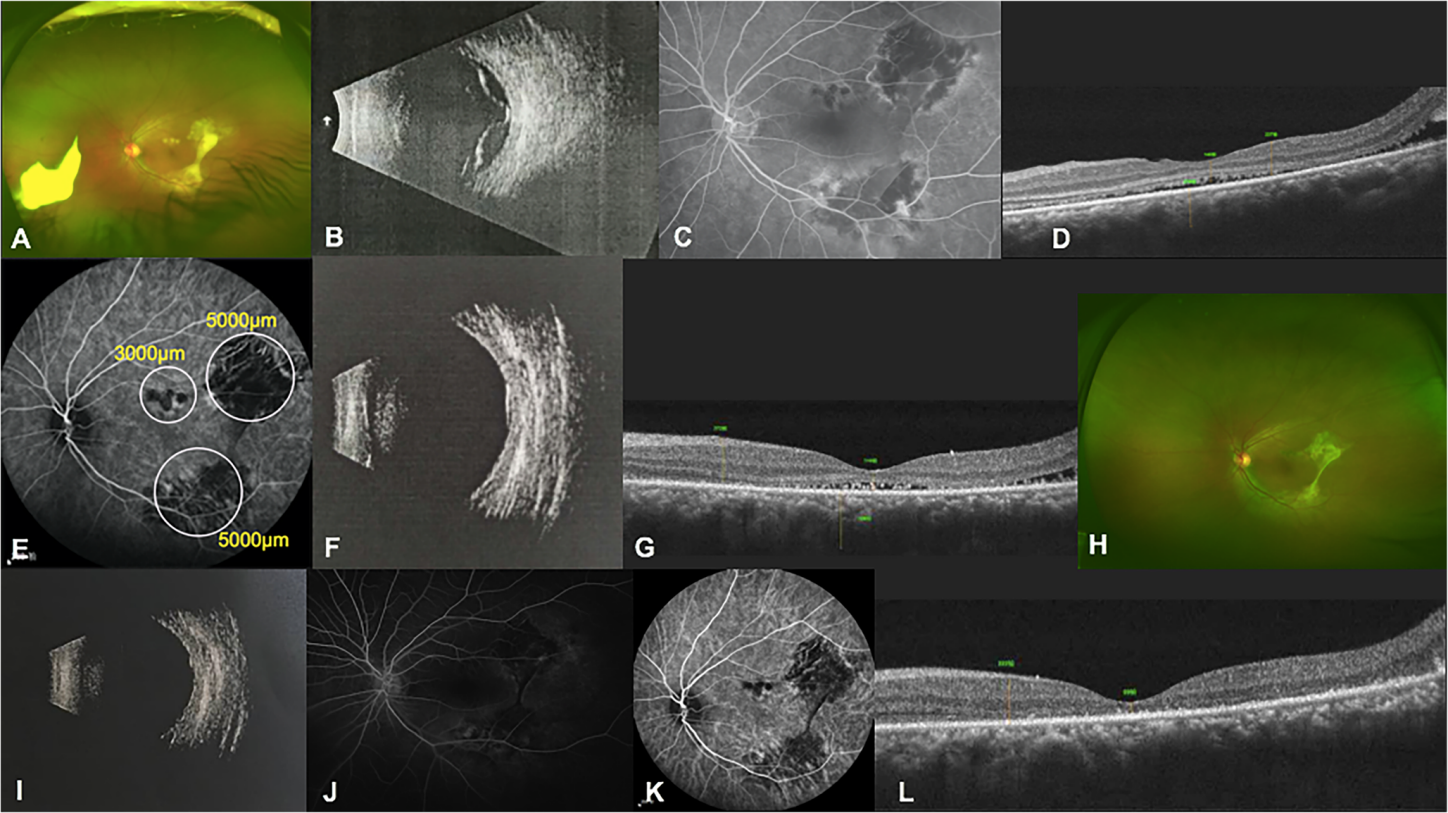
Clinical examinations of the left eye in case 4. **a** Fundus photograph illustrating TA in the nasal retina and non-rhegmatogenous retinal detachment with multifocal exudative lesions in the posterior pole at baseline. **b** Ophthalmic B scan revealing bullous retinal detachment at baseline. **c** FFA image demonstrating hyperfluorescent leakages in areas corresponding to exudative retinal detachment at baseline. **d** OCT image showing SRF in the macular and temporal retinal areas at baseline. **e** ICGA image illustrating hyperfluorescent leakages of the choroid around the lesion area at baseline. White circles indicate the spot sizes of PDT. **f** Ophthalmic B scan revealing bullous retinal detachment partly resolved 1 month after PDT. **g** OCT image revealing reduced SRF 1 month after PDT. **h** Fundus photograph revealing the disappearance of TA and bullous retinal detachment 3 months after PDT. **i** Ophthalmic B scan revealing the reattachment of the retina 3 months after PDT. **j** FFA image showing hypofluorescence in the lesion area 3 months after PDT. **k** ICGA image showing hypofluorescence in the lesion area 3 months after PDT. **l** OCT image showing the disappearance of SRF 3 months after PDT

A 48-year-old man presented with blurred vision in the left eye for 2 weeks. He had a history of intravitreal injection of triamcinolone acetonide (TA) for a misdiagnosis of uveitis in the left eye 1 month prior to examination. Additionally, his left eye was treated with two periocular injections of dexamethasone, one intravitreal injection of conbercept and one application of laser photocoagulation within 1 month at other hospital. However, there was with no improvement of visual acuity. His BCVA was 20/20 in the right eye and 20/200 in the left when he came to our outpatient facility. Ophthalmoscopy of the left eye disclosed intraocular TA in the nasal retina and non-rhegmatogenous retinal detachment with multifocal exudative lesions in the posterior pole (Fig. 5a). An ophthalmic B scan revealed retinal detachment in the left eye (Fig. 5b). FFA showed multiple hyperfluorescent leakage in areas corresponding to exudative retinal detachment at baseline (Fig. 5c). OCT disclosed SRF at the area of macula and temporal retina (Fig. 5d). His left eye received a 50% dose of verteporfin PDT with two spots of 5000 μm and one spot of 3000 μm under the guidance of ICGA (Fig. 5e). One month later, an ophthalmic B scan revealed obviously reduced bullous retinal detachment, and OCT disclosed the decreased SRF (Fig. 5f, g). Three months after PDT, ophthalmoscopy and an ophthalmic B scan revealed the completely resolved exudative retinal detachment (Fig. 5h, i). FFA showed hypofluorescence, and ICGA revealed hypofluorescence in the lesion area (Fig. 5j, k). SRF on the OCT image disappeared, and the macula regained normal anatomic structure (Fig. 5l). At the 6-month examination, his vision acuity improved to 20/100 in the left eye, and no recurrence occurred.

#### Case 5

**Fig. 6:**
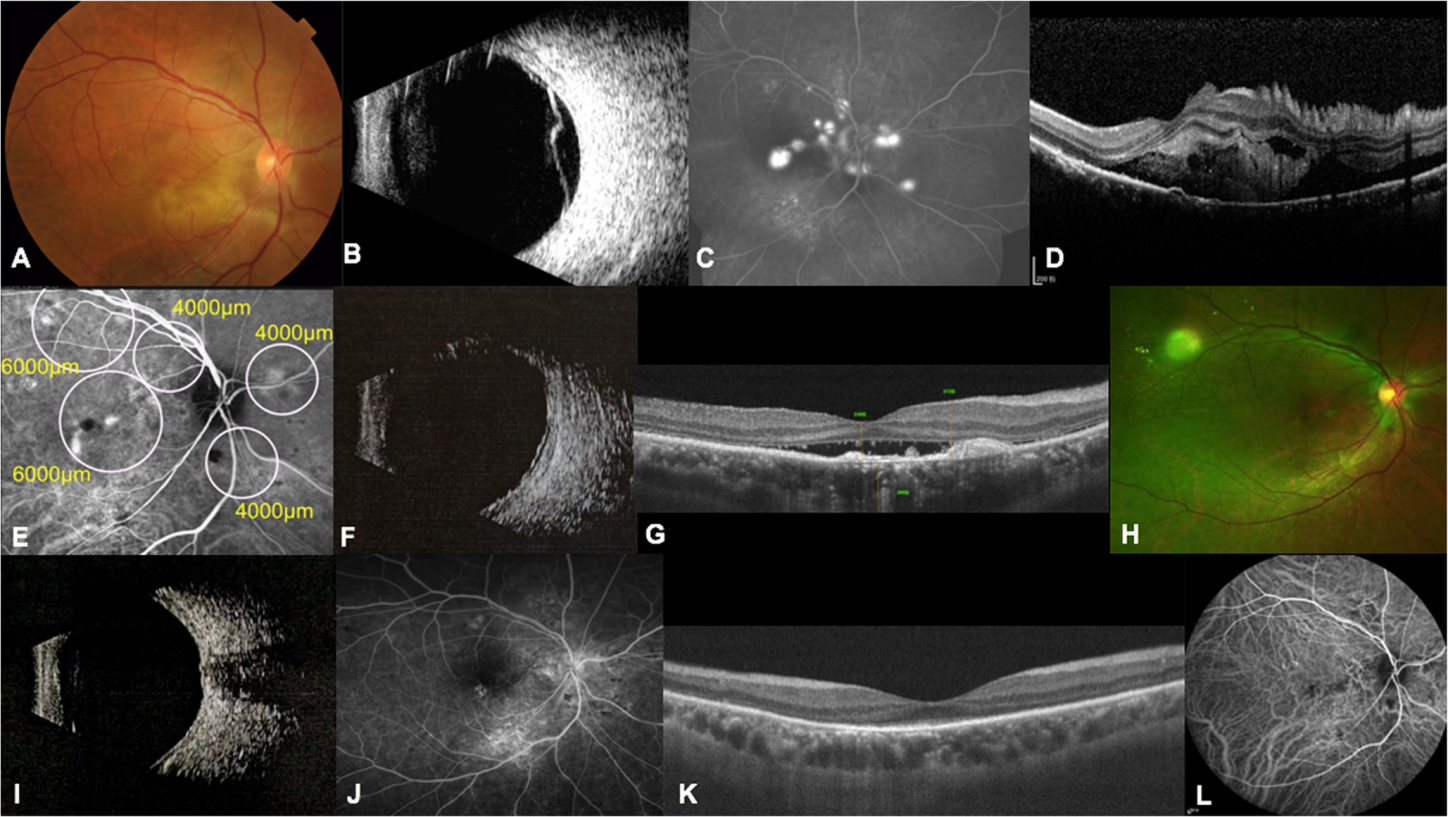
Clinical examinations of the right eye in patient 5. **a** Fundus photograph taken at the initial presentation illustrating bullous retinal detachment. **b** Ophthalmic B scan confirming the bullous retinal detachment. **c** FFA image demonstrating several hyperfluorescent leakages around the optic disc corresponding to exudative lesions at baseline. **d** OCT image showing exudative retinal detachment with serious SRF and sub-retinal fibrin. Retinal folds can be revealed at baseline. **e** ICGA image illustrating hyperfluorescent leakages of the choroid at baseline. White circles indicate the spot sizes of PDT. **f** Ophthalmic B scan revealing an obvious reduction in bullous retinal detachment 1 month after PDT. **g** OCT image revealing decreases in SRF and sub-retinal fibrin and the disappearance of retinal folds 1 month after PDT. **h** Fundus photograph illustrating the disappearance of bullous retinal detachment 3 months after PDT. **i** Ophthalmic B scan revealing the reattachment of the retina 3 months after PDT. **j** FFA image showing the mottled shape detected by fluorescence transmission and no fluorescein leakage 3 months after PDT. **k** OCT image showing the complete resolution of SRF and sub-retinal fibrin 3 months after PDT. **l** ICGA image showing hypofluorescence in the lesion area 3 months after PDT

A 50-year-old man presented with blurred vision in the right eye for a year. His BCVA was 20/200 in the right eye, while the left eye worsened to no light perception because of glaucoma at an early age. Ophthalmoscopy of the right eye disclosed non-rhegmatogenous retinal detachment in the posterior pole, and ophthalmic B scan confirmed the bullous retinal detachment (Fig. 6a, b). FFA revealed several hyperfluorescent leakages around the optic disc corresponding to exudative lesions (Fig. 6c). OCT of the right eye disclosed exudative retinal detachment with serious SRF, sub-retinal fibrin, and retinal folds (Fig. 6d). Five spots of 50% dose of verteporfin PDT were administered under the guidance of ICGA (Fig. 6e). One month after PDT, an ophthalmic B scan revealed obviously reduced bullous retinal detachment (Fig. 6f). SRF and sub-retinal fibrin decreased and the retinal folds disappeared from the OCT image (Fig. 6g). Three months after PDT, ophthalmoscopy and an ophthalmic B scan revealed that the bullous retinal detachment was completely resolved (Fig. 6h, i). A mottled shape was observed by fluorescence transmission, and no fluorescein leakage was found in the FFA image (Fig. 6j). OCT showed normal macula structure in the right eye (Fig. 6k). ICGA disclosed hypofluorescence in the lesion area (Fig. 6l). His BCVA improved to 20/40, with no recurrence during the follow-up of more than 6 months.

#### Case 6

**Fig. 7:**
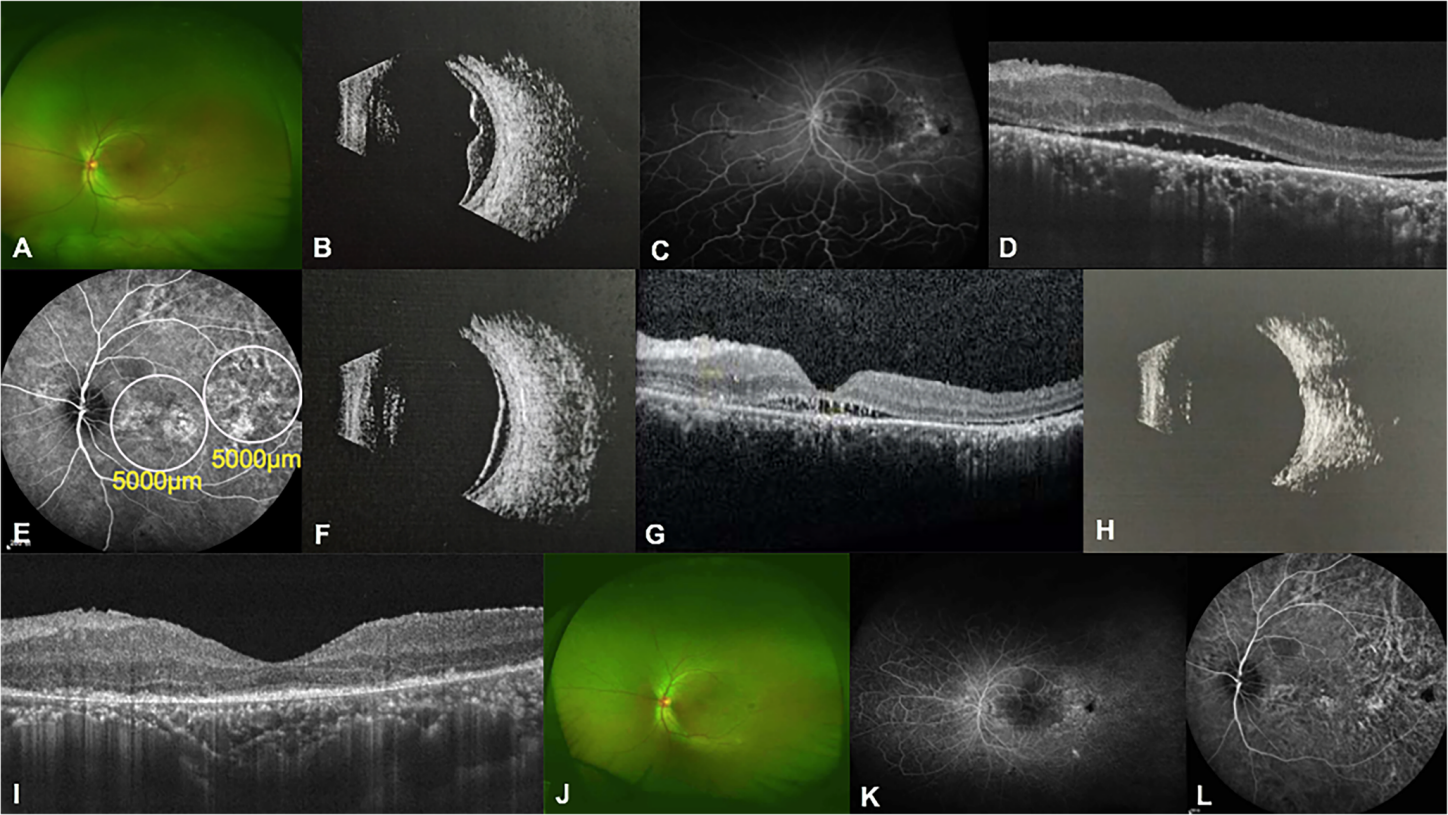
Clinical examinations of the left eye in patient 6. **a** Fundus photograph taken at the initial presentation illustrating bullous retinal detachment in the inferior retina. **b** Ophthalmic B scan confirming the bullous retinal detachment at baseline. **c** FFA image demonstrating hyperfluorescent leakages in temporal retinal areas and hypofluorescent exudative retinal lesions corresponding to retinal detachment in the inferior retina at baseline. **d** OCT image disclosing exudative retinal detachment with serious SRF at baseline. **e** ICGA image illustrating hyperfluorescent leakages of the choroid at baseline. White circles indicate the spot sizes of PDT. **f** Ophthalmic B scan revealing a reduction in bullous retinal detachment 1 month after PDT. **g** OCT image revealing a decrease in SRF 1 month after PDT. **h** Ophthalmic B scan revealing the reattachment of the retina 3 months after PDT. **i** OCT image showing the complete resolution of SRF 3 months after PDT. **j** Fundus photograph illustrating a normal fundus 3 months after PDT. **k** FFA image showing a mottled shape in fluorescence transmission and no fluorescein leakage 6 months after PDT. **l** ICGA image showing hypofluorescence in the lesion area 6 months after PDT

A 35-year-old man presented with blurred vision in the left eye for near 3 months. His BCVA was 20/25 in the right eye and 20/800 in the left eye. Ophthalmoscopy of the left eye revealed retinal detachment in the inferior retina (Fig. 7a). An ophthalmic B scan confirmed bullous retinal detachment in the left eye (Fig. 7b). FFA disclosed multiple hyperfluorescent leakages in areas of temporal retina and hypofluorescent exudative retinal lesions corresponding to retinal detachment in the inferior retina (Fig. 7c). OCT disclosed large amounts of SRF (Fig. 7d). His left eye received a 50% dose of verteporfin PDT with two spots of 5000 μm under the guidance of ICGA (Fig. 7e). One month after PDT, the SRF was partly resolved based on an ophthalmic B scan and OCT (Fig. 7f, g). Three months after PDT, SRF was completely resolved, as confirmed by ophthalmic B scan, OCT, and ophthalmoscopy (Fig. 7h–j). At the 6-month examination after photodynamic therapy, his BCVA improved to 20/80 in the left eye. An FFA image showed a mottled shape in fluorescence transmission, and no fluorescein leakage was found (Fig. 7k). An ICGA image showed the disappearance of hyperfluorescence leakage in the lesion area (Fig. 7l).

## Discussion

In this study, we evaluated the efficacy of half-dose verteporfin PDT with multifocal and large laser spots in the treatment of bullous retinal detachment. Although PDT is generally considered a common therapy for classic CSC [10], it has not been popularized among the treatment of bullous retinal detachment, a severe variant of CSC.

Besides PDT, studies suggested that laser photocoagulation may be of benefit in reducing SRF and oral administration of mineralocorticoid receptor antagonist eplerenone or intravitreal antiangiogenic drugs may be helpful to improve visual acuity [11, 12]. Some cases also indicated that transpupillary thermotherapy seemed to be effective for the treatment of bullous variant of CSC in the short term and internal drainage could lead to anatomical and functional improvement [13, 14]. In fact, the treatment for bullous retinal detachment has not been well established. In past decades, laser photocoagulation was the traditional treatment for extrafoveal leakage area [15]. The rapid resolution of bullous retinal detachment was reported after photocoagulation of the areas of RPE detachment [16]. However, there have been only a limited number of case reports on the use of PDT for bullous retinal detachment [17, 18].

In 1973, Gass first reported a group of patients with bullous exudative detachment [19]. Bullous retinal detachment is a rare sub-type of CSC characterized by multifocal posterior exudations with shifting SRF [20]. CSC is a common maculopathy mainly affecting working-age populations and is observed more frequently in men than in women [21]. In a previous study, Balaratnasingam et al. [9] reported a group of bullous retinal detachment patients with a mean age of 53.8 years, and 50% of patients were bilaterally affected, while Otsuka et al. [22] reported 25 patients—21 men and 4 women—with a mean age at disease onset of 43.1 years. Our study showed that the mean age was 40.5 ± 6.7 years and the male-to-female ratio was 5:1, which was similar to previous reports. However, the disease affected a single eye in all six patients, accounting for 100% in our group, which was different from the results of previous studies [9, 23].

Previous studies have shown an association between retinal disease and retinal or cardiac transplantation with the bullous variant of CSC [24, 25]. Corticosteroids also have been implicated in the pathogenesis of bullous retinal detachment [7, 26]. Balaratnasingam et al. [9] reported that 50% of patients had corticosteroid use in the bullous retinal detachment group. In the present study, two patients, accounting for 33.3%, had undergone corticosteroid therapy: one (patient 1) for initial disease of nephritic syndrome and the other (patient 4) for intravitreal injection of TA. The latter patient was initially misdiagnosed with uveitis by another ophthalmologist and was subsequently administered various treatments, including corticosteroids, prior to visiting our hospital. Although CSC is a self-limiting condition in the majority of patients, the recognition of this atypical form is important. Failure to differentiate this condition from inflammatory disease may result in the inappropriate use of corticosteroids, leading to disease progression and visual loss. This disease can also occur spontaneously without any history of corticosteroid use. The remaining four patients, accounting for 66.7% in the present case series, initially presented with characteristics of classic CSC, followed by the development of bullous retinal detachment 3 months to 10 years later. A previous study reported that patients with classic CSC developed the severe variant after 7 months to 9 years [20]. A patient in the present study progressed to bullous retinal detachment in the shortest time of 3 months from the stage of acute CSC, which, to our knowledge, has seldom been reported. The new findings in the present study will aid the exploration of the pathophysiology of bullous retinal detachment. We suggest that bullous retinal detachment is not a single pathogenic risk factor but may be a result of interactions between environmental, genetic, and individual conditions.

Peripheral hyperfluorescent foci were observed on FFA in 83.3% of eyes in the present study. We also observed sub-retinal fibrin on OCT in 50% of affected eyes. Hooymans [27] reported fibrotic scar formation in the development of CSC to severe variants of bullous retinal detachment during systematic treatment with corticosteroids. Balaratnasingam et al. described a more frequent occurrence of sub-retinal fibrin in the eyes of the bullous CSC group than in the eyes of the non-bullous CSC group [9]. Retinal folds were observed in 33.3% of eyes with bullous retinal detachment in the present case series. We inferred the folds were association with retinal edema and choroidal vasodilatation.

In the present case series, all affected eyes received half-dose PDT, including five eyes that received single PDT, accounting for 83.3%, and one eye that received a second PDT, accounting for 16.7%. The second PDT was administered when SRF accumulation was not reduced until 2 months after the first therapy. After adjustment of the therapeutic area guided by ICGA, retinal reattachment was acquired 1 month later. Studies have shown that vascular damage and cytotoxicity associated with PDT are dosage-dependent [28]. Considering the dosage-dependent administration of verteporfin and the results of a previous study on the efficacy of PDT in the treatment of CSC, we used half-dose PDT in the present study [29]. The PDT treatment was guided by ICGA, which has become the mainstream procedure to guide PDT to target hyperfluorescent areas in mid-phase [30, 31]. The entire area of abnormal choroidal vessels could be covered by ICGA-guided PDT to prevent further leakage. The spot sizes in the present study ranged from 3000 to 6000 μm with a mean size of 4647 ± 996 μm in diameter.

The success rate of half-dose PDT in the present study was defined as the reattachment of retina on ophthalmology B scan. These data showed a success rate of 100% after PDT treatment for bullous retinal detachment, with an absence of SRF on OCT. The recurrence rate was as low as zero during the follow-up of more than 6 months. Remarkably, the visual results were significantly improved 3 and 6 months after half-dose PDT compared to the baseline. Similar results in case series of bullous retinal detachment have not previously been reported. The present study provides clinical evidence for the treatment of bullous retinal detachment with PDT.

Since the present sample size was small and the follow-up time was short, a prospective randomized control study involving a large number of patients with long-term follow-up is needed for further investigation of the advantage of PDT.

In conclusion, this study has demonstrated the effective treatment of half-dose PDT with multifocal and large laser spots in bullous retinal detachment, leading to significant visual and anatomic improvement in all affected eyes in the present case series. We are currently performing a multicenter prospective randomized controlled trial to explore the efficacy of PDT for bullous retinal detachment.
